# Unconventional superconductivity in topological Kramers nodal-line semimetals

**DOI:** 10.1126/sciadv.abq6589

**Published:** 2022-10-28

**Authors:** Tian Shang, Jianzhou Zhao, Lun-Hui Hu, Junzhang Ma, Dariusz Jakub Gawryluk, Xiaoyan Zhu, Hui Zhang, Zhixuan Zhen, Bocheng Yu, Yang Xu, Qingfan Zhan, Ekaterina Pomjakushina, Ming Shi, Toni Shiroka

**Affiliations:** ^1^ Key Laboratory of Polar Materials and Devices (MOE), School of Physics and Electronic Science, East China Normal University, Shanghai 200241, China.; ^2^ Co-Innovation Center for New Energetic Materials, Southwest University of Science and Technology, Mianyang 621010, China.; ^3^ Department of Physics and Astronomy, University of Tennessee, Knoxville, TN 37996, USA.; ^4^ Department of Physics, City University of Hong Kong, Kowloon, Hong Kong.; ^5^ Laboratory for Multiscale Materials Experiments, Paul Scherrer Institut, CH-5232 Villigen PSI, Switzerland.; ^6^ Swiss Light Source, Paul Scherrer Institut, CH-5232 Villigen PSI, Switzerland.; ^7^ Laboratory for Muon-Spin Spectroscopy, Paul Scherrer Institut, CH-5232 Villigen PSI, Switzerland.; ^8^ Laboratorium für Festkörperphysik, ETH Zürich, CH-8093 Zürich, Switzerland.

## Abstract

Crystalline symmetry is a defining factor of the electronic band topology in solids, where many-body interactions often induce a spontaneous breaking of symmetry. Superconductors lacking an inversion center are among the best systems to study such effects or even to achieve topological superconductivity. Here, we demonstrate that *T*RuSi materials (with *T* a transition metal) belong to this class. Their bulk normal states behave as three-dimensional Kramers nodal-line semimetals, characterized by large antisymmetric spin-orbit couplings and by hourglass-like dispersions. Our muon-spin spectroscopy measurements show that certain *T*RuSi compounds spontaneously break the time-reversal symmetry at the superconducting transition, while unexpectedly showing a fully gapped superconductivity. Their unconventional behavior is consistent with a unitary (*s* + *ip*) pairing, reflecting a mixture of spin singlets and spin triplets. By combining an intrinsic time-reversal symmetry-breaking superconductivity with nontrivial electronic bands, *T*RuSi compounds provide an ideal platform for investigating the rich interplay between unconventional superconductivity and the exotic properties of Kramers nodal-line/hourglass fermions.

## Teaser

Broken time-reversal symmetry ruthenium silicides that host Kramers- and hourglass fermions promise topological superconductivity.

## INTRODUCTION

The effects of many-body interactions and spin-orbit coupling (SOC) in crystalline symmetry–enforced semimetals are a topical question in condensed matter physics. In particular, materials that lack an inversion center are among the best candidates for studying topological phenomena (*1*–*7*), since noncentrosymmetric materials with a nonsymmorphic symmetry can generate exotic types of fermionic excitations. For instance, Weyl fermions, originally envisioned in high-energy physics (*8*), were experimentally found as quasiparticles in Ta(As,P) and Nb(As,P) (*9*–*12*). The topological electronic properties of nonmagnetic crystals with SOC have also been investigated theoretically. Thus, Kramers Weyl fermions pinned at time-reversal–invariant momenta were identified in materials with chiral space groups (*13*), while Kramers nodal-line (KNL) fermions were forecasted to occur in noncentrosymmetric metals (*14*). Akin to the original Kramers theorem, which predicts at least doubly degenerate energy levels in half-integer spin systems (i.e., with an odd total number of fermions), here, we have doubly degenerate lines (called KNLs), connecting time-reversal–invariant momenta. In addition, noncentrosymmetric materials may also host exotic fermions with an hourglass-shaped dispersion, protected by glide reflection (*15*–*17*) and known to exhibit interesting topological properties, intimately related to the space group symmetry. Hourglass fermions were originally predicted to occur on the surface of nonsymmorphic KHgSb crystals and later on experimentally confirmed by angle-resolved photoemission spectroscopy (ARPES) (*15*, *18*).

To date, intense research on semimetals has been primarily focused on their noninteracting electronic band topology. Many noncentrosymmetric materials are now known to be topological semimetals (*1*–*7*), which, because of their great potential for applications, are at the forefront of quantum matter and materials science research. On the contrary, the interplay between topology and correlated electronic states, such as unconventional superconductivity (SC) or magnetism, remains largely unexplored in semimetals. Although topological semimetals that show unconventional SC are promising materials for realizing topological SC, they are extremely rare and, hence, have been overlooked.

In parallel, current research has shown that several noncentrosymmetric materials exhibit SC at low temperatures (*19*–*21*) and, in view of their structure, are known as noncentrosymmetric superconductors (NCSCs). Time-reversal symmetry (TRS) breaking has been shown to occur in the superconducting state of several NCSCs (*22*–*30*). Broken TRS in the superconducting state, one of the possible realizations of unconventional SC, is here manifested as the spontaneous appearance of magnetic fields below the onset of SC. Whenever available, NCSCs with broken TRS represent an ideal platform to study the rich interplay between the exotic properties of topological states and unconventional SC. The possibility to host unconventional and topological SC, or to act as systems where to realize the Majorana zero modes, has made NCSCs one of the most investigated superconducting families (*31*, *32*).

Here, we combine muon-spin relaxation and rotation (μSR) measurements with theoretical band structure calculations and symmetry analyses to identify *T*RuSi compounds (with *T* a transition metal) as a unique family of NCSCs. They not only spontaneously break TRS at the superconducting transition and exhibit a fully gapped SC state with a mixed singlet and triplet pairing but also serve as remarkable materials for investigating various topological states, including Kramers and hourglass fermions, as well as their interplay with unconventional SC. Consequently, the *T*RuSi family represents one of the best candidates for hosting topological SC, with potential applications in quantum computation.

## RESULTS

### Crystal structure and SC

We consider first the interplay between topology and electronic correlations in the ruthenium-based ternary silicides *T*RuSi, where *T* is a transition or a rare-earth metal. The *T*RuSi class of materials features several distinct structural symmetries, such as tetragonal PbClF-type (*P*4/*nmmZ*, no. 129) (*33*), orthorhombic TiNiSi-type (*Pnma*, no. 62) (*34*), hexagonal ZrNiAl type ($P\bar{6}2m$, no. 189) (*35*), and orthorhombic TiFeSi-type (*Ima*2, no. 46) (*35*). While the former two structures are often encountered in rare-earth ruthenium silicides, the latter two are usually found in their transition metal counterparts. Among the four different symmetries, the ZrNiAl- and TiFeSi-type structures belong to the noncentrosymmetric class and lack an inversion center. Several ZrNiAl-type compounds are known to exhibit SC (*36*–*39*). However, most of them are ordinary fully gapped superconductors with preserved TRS and, hence, are conventional superconductors (*38*, *40*). In the *T*RuSi case (with *T* = Ti, Nb, Hf, and Ta), all the compounds adopt a TiFeSi-type noncentrosymmetric structure (*35*, *41*, *42*), with both NbRuSi and TaRuSi becoming superconductors below ∼4 K (*35*, *43*). To date, their superconducting properties remain largely unexplored.

**Fig. 1.**
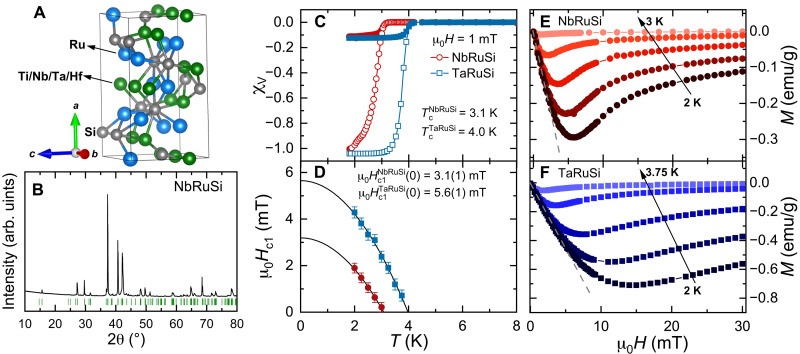
Crystal structure and SC. (**A**) The orthorhombic crystal structure (unit cell) of *T*RuSi (*T* = Ti, Nb, Hf, and Ta). (**B**) Room temperature powder XRD patterns for NbRuSi. The vertical bars mark the Bragg peak positions corresponding to the *Ima*2 space group. The other *T*RuSi samples show similar XRD patterns. (**C**) Temperature dependence of magnetic susceptibility, measured in a field of μ_0_*H* = 1 mT, using both field-cooled (FC) and zero-field–cooled (ZFC) protocols. The well-distinct ZFC and FC curves are consistent with type II SC, as confirmed also by μSR measurements. (**D**) Lower critical fields *H*_c1_ versus temperature. Solid lines are fits to μ_0_*H*_c1_(*T*) = μ_0_*H*_c1_(0)[1 − (*T*/*T_c_*)^2^]. (**E** and **F**) Field-dependent magnetization recorded at various temperatures for NbRuSi (E) and TaRuSi (F). For each temperature, *H*_c1_ was determined as the value where *M*(*H*) starts deviating from linearity (indicated by dashed lines). Error bars of *H*_c1_ coincide with the field steps in the field-dependent magnetization measurements. Both the magnetic susceptibility and lower critical field were corrected by the demagnetization factors obtained from the field-dependent magnetization at 2 K.

We synthesized a series of *T*RuSi compounds and systematically investigated their physical properties via magnetic susceptibility, heat capacity, electrical resistivity, and μSR measurements, as well as via theoretical calculations and symmetry analyses. As shown in Fig. 1A, *T*RuSi compounds crystallize in a TiFeSi-type noncentrosymmetric orthorhombic structure with a *C*_2*v*_ point group, as confirmed by the powder x-ray diffraction (XRD) patterns in Fig. 1B. While TiRuSi behaves as a normal metal, without undergoing any phase transitions, and HfRuSi exhibits only a marginal SC fraction (see figs. S1 to S3), NbRuSi and TaRuSi both show bulk SC. The magnetic susceptibilities in Fig. 1C indicate clear SC transitions at *T*_c_ = 3.1 and 4.0 K in NbRuSi and TaRuSi, consistent with the values determined from electrical resistivity and heat capacity measurements (see figs. S1 and S4). Both compounds show a full diamagnetic screening, an indication of bulk SC, further confirmed by a prominent specific heat jump at *T*_c_. In both cases, the lower critical fields *H*_c1_ versus temperature are summarized in Fig. 1D. The *H*_c1_(*T*) curves, as determined from the field-dependent magnetization data *M*(*H*) (see Fig. 1, E and F), provide μ_0_H_c1_(0) = 3.1(1) and 5.6(1) mT for NbRuSi and TaRuSi, respectively.

### ZF-μSR and TRS breaking

**Fig. 2.**
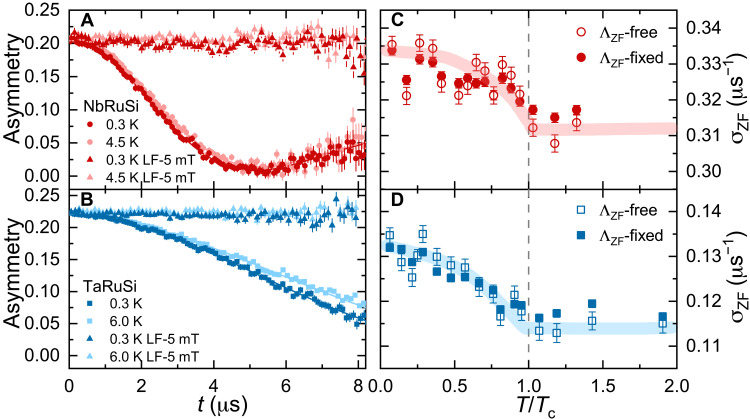
ZF-μSR and evidence of TRS breaking. (**A** and **B**) Representative ZF-μSR spectra collected above and below *T*_c_ for NbRuSi (A) and TaRuSi (B). The flat μSR datasets correspond to LF-μSR spectra collected in a 5-mT LF, suggesting that the muon spins are easily decoupled from the local field, even in case of a small LF. Solid lines through the data are fits to Eq. 6. (**C** and **D**) ZF muon-spin relaxation rate σ_ZF_ versus the reduced temperature *T*/*T*_c_ for NbRuSi (C) and TaRuSi (D). The σ_ZF_(*T*) values, derived from either a free- or a fixed-Λ_ZF_ analysis, are presented. The consistent increase of σ_ZF_ below *T*_c_ reflects the onset of spontaneous magnetic fields, indicative of a breaking of TRS in the superconducting state, while the Λ_ZF_(*T*) is practically independent of temperature (see details in fig. S5). The dashed line marks the ZF *T*_c_ value. The error bars of σ_ZF_ are the SDs obtained from fits to Eq. 6 by the musrfit software package (*76*).

To date, there are mostly two direct ways to probe superconducting states with a spontaneously broken TRS: zero-field (ZF) μSR (*22*–*30*, *44*, *45*) and polar Kerr effect (*46*, *47*). ZF-μSR is extremely sensitive to small changes in the local magnetic field (with a resolution down to ∼0.01 mT) (*48*). Hence, a possible increase in the ZF muon-spin relaxation rate below the onset of SC provides direct evidence for superconducting states with broken TRS. To search for evidence of TRS breaking in the NbRuSi and TaRuSi superconductors, we performed ZF-μSR measurements at various temperatures, covering their normal and superconducting states, with representative ZF-μSR spectra being shown in Fig. 2 (A and B). The different asymmetry curves (i.e., muon-spin relaxations) of NbRuSi and TaRuSi reflect the substantial different nuclear magnetic moments of Ta (2.37 μ_n_) and Nb (6.17 μ_n_) atoms, the latter justifying the threefold higher ZF relaxation rate of NbRuSi above *T*_c_. The ZF-μSR spectra of NbRuSi exhibit small yet clear differences between 0.3 K and temperatures above *T*_c_ (e.g., 4.5 K; see Fig. 2A). As expected, these are more evident in TaRuSi (see Fig. 2B), in view of its lower normal-state relaxation rate.

Since both NbRuSi and TaRuSi are nonmagnetic, in the absence of external magnetic fields, their muon-spin relaxation is generally described by a phenomenological relaxation function, consisting of a combination of Gaussian and Lorentzian Kubo-Toyabe relaxations (see Eq. 6) (*49*, *50*). Since the Lorentzian relaxation rates Λ_ZF_(*T*) exhibit an almost temperature-independent behavior in the studied temperature range (see fig. S5), the Gaussian relaxation rates σ_ZF_ were estimated by fixing Λ_ZF_ to their average value ($\Lambda_{\text{ZF}}^{\text{avg}.}$ = 0.005 and 0.010 μs^−1^ for NbReSi and TaRuSi). In both cases, σ_ZF_(*T*) exhibits an evident increase below the onset of SC (Fig. 2, C and D). Conversely, σ_ZF_(*T*) is almost flat in the normal state, thus excluding a possible origin related to magnetic fluctuations or impurities, typically leading to a Curie-Weiss behavior in the muon-spin relaxation rates [i.e., σ_ZF_(*T*) or Λ_ZF_(*T*) ∝ *T*^−1^] (*51*). Both free- and fixed-Λ_ZF_ analyses show a robust increase in σ_ZF_(*T*) below *T*_c_, further confirming that the signal of spontaneous magnetic fields is an intrinsic effect. Very recently, theoretical works have confirmed the intrinsic nature of spontaneous magnetic fields in superconductors with TRS breaking (*52*). The ground-state spontaneous fields can be estimated by using $B_{\text{spont.}}=\sqrt{2}\Delta \sigma_{\text{ZF}}/\gamma_{\mu }$, where Δσ_ZF_ represents the change of σ_ZF_ between the normal and superconducting states. The estimated *B*_spont._ ≈ 0.040 and 0.035 mT for NbRuSi and TaRuSi are similar to those of other TRS-breaking superconductors, as e.g., α-Mn–type NCSCs (*25*, *26*).

Last, longitudinal-field (LF) μSR spectra (flat datasets in Fig. 2, A and B), collected in the superconducting and the normal states of both compounds, indicate that a field of only 5 mT is sufficient to fully decouple the muon spins from the fields responsible for the TRS-breaking relaxation channel. Once more, the LF-μSR results rule out an extrinsic origin (e.g., magnetic impurity induced) of the additional relaxation in σ_ZF_(*T*). Considered together, the ZF- and LF-μSR results reveal that the enhanced σ_ZF_(*T*) below *T*_c_ provides clear evidence of the occurrence of spontaneous magnetic fields, in turn, the signature of broken TRS in the superconducting state of NbRuSi and TaRuSi.

### TF-μSR and fully gapped SC

**Fig. 3.**
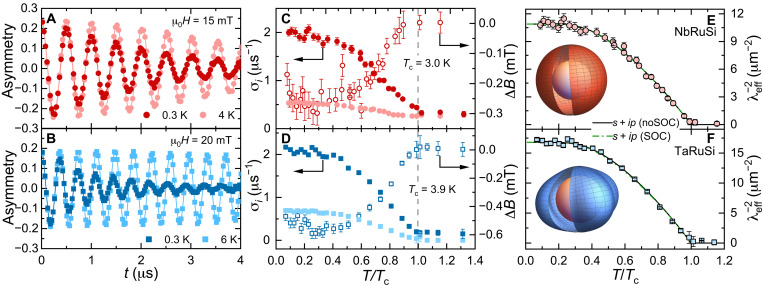
TF-μSR and superfluid density. (**A** and **B**) TF-μSR spectra collected in the superconducting and normal states (i.e., above and below *T*_c_) of NbRuSi (A) and TaRuSi (B). (**C** and **D**) Muon-spin relaxation rate σ*_i_*(*T*) (left axis) and diamagnetic shift Δ*B*(*T*) (right axis) versus the reduced temperature *T*/*T*_c_ for NbRuSi (C) and TaRuSi (D). As indicated by the dashed line, a diamagnetic field shift appears below *T*_c_ and is attributed to the formation of the FLL. (**E** and **F**) Superfluid density [$\rho_{\text{sc}}(T)\propto \lambda_{\text{eff}}^{-2}(T)$] as a function of reduced temperature *T*/*T*_c_ for NbRuSi (E) and TaRuSi (F). Solid and dash-dotted lines represent fits to Eq. 7 with a nonsymmetric (*s* + *ip*) pairing in the weak and strong SOC limit, with the gap functions shown in the insets of (E) and (F) (see details in the text). The error bars of σ*_i_*(*T*), Δ*B*(*T*), and $\lambda_{\text{eff}}^{-2}(T)$ are the SDs obtained from fits of the TF-μSR spectra to Eq. 2 by the musrfit software package (*76*).

To investigate at a microscopic level the superconducting pairing in NbRuSi and TaRuSi, their temperature-dependent magnetic penetration depth was determined via transverse-field (TF) μSR measurements. Since the superfluid density ρ_sc_ is proportional to the inverse square of the magnetic penetration depth (i.e., ρ_sc_ $\propto \lambda_{\text{eff}}^{-2}$), the measured temperature-dependent λ_eff_(*T*) reveals the nature of superconducting pairing (see Materials and Methods). As illustrated in Fig. 3 (A and B), the TF-μSR measurements were performed in an applied field of 15 and 20 mT for NbRuSi and TaRuSi, covering both the normal and superconducting states. The development of a flux-line lattice (FLL) in the mixed state broadens the internal field distribution (see fig. S6) and leads to an enhanced muon-spin relaxation rate. As shown in Fig. 3 (C and D), below *T*_c_, the relaxation rates start to increase due to the onset of FLL and the increased superfluid density. Simultaneously, a diamagnetic field shift Δ*B* appears below *T*_c_. The inverse square of the effective magnetic penetration depth $\lambda_{\text{eff}}^{-2}(T)$ versus the reduced temperature *T*/*T*_c_ is presented in Fig. 3 (E and F) for NbRuSi and TaRuSi, respectively. The temperature-invariant superfluid density for *T* < *T*_c_/3 clearly hints at the absence of low-energy excitations and, hence, suggests a fully gapped superconducting state in both NbRuSi and TaRuSi. The ρ_sc_(*T*) is well described by a nonsymmetric (*s* + *ip*) pairing in both the weak (solid line) and the strong SOC limit (dash-dotted line) across the full temperature range (here, the weak SOC limit is identical to the *s*-wave pairing; see details in Discussion). However, only the strong SOC limit is consistent with a broken TRS superconducting state in *T*RuSi (see Fig. 2). The fit parameters for both compounds are listed in table S1. Since the electronic bands of both compounds show multiband features, we also attempted to analyze ρ_sc_(*T*) with a two-gap *s*-wave model. However, such two-gap fit was virtually indistinguishable from the one-gap fit (see fig. S7 and table S1). This might be attributed to the relatively small weight of the second gap or to the comparable gap sizes, either factor making it difficult to discriminate between a single- and a two-gap superconductor based on the temperature-dependent superfluid density alone, as already reported in other compounds (*53*, *54*).

### Electronic band structure and topology

**Fig. 4.**
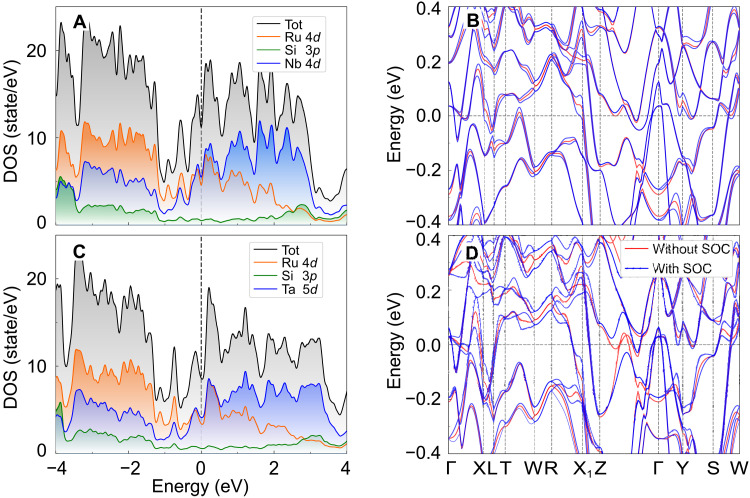
Electronic band structure. (**A** and **B**) The calculated DOS (A) and electronic band structures (B) for NbRuSi. (**C** and **D**) The analogous results for TaRuSi. The band structures in (B) and (D) were calculated by ignoring and by considering the SOC, corresponding to the red and blue lines, respectively. The DOS and the electronic band structures of TiRuSi and HfRuSi are presented in fig. S8.

To gain further insight into the normal and superconducting state properties of *T*RuSi, we performed detailed electronic band structure calculations via density functional theory (DFT). Close to the Fermi level (*E*_F_), the density of states (DOS) is dominated by Nb-4*d* (Ti-3*d*, Hf-5*d*, and Ta-5*d*) and Ru-4*d* orbitals, while the contribution from Si-3*p* orbitals is generally negligible (see Fig. 4, A and C, and fig. S8). Only below −1 eV from *E*_F_ the Si-3*p* orbitals start to affect the low-lying energy states. The electronic band structures of NbRuSi and TaRuSi, calculated by ignoring and by considering the SOC, are shown in Fig. 4 (B and D), respectively (see fig. S8 for TiRuSi and HfRuSi). Up to five (three) bands are identified to cross the Fermi level of NbRuSi (TaRuSi), confirming their multiband nature (see fig. S9). TiRuSi and HfRuSi, too, exhibit multiband features. Because of the lifting of degeneracy once SOC is taken into account, the bands separate, with one of them ending up closer to the Fermi level. For instance, in NbRuSi, near the *X* (*X*_1_) and *Y* points, the band splitting *E*_ASOC_ caused by antisymmetric SOC (ASOC) is significant. The estimated band splittings in NbRuSi and TaRuSi are *E*_ASOC_ ∼ 100 and 300 meV, equivalent to *E*_ASOC_/*k*_B_*T*_c_ ∼ 385 and 882, respectively. Although smaller than the band splitting in CePt_3_Si (*55*), they are comparable to that of CaPtAs and Li_2_Pt_3_B (*29*, *56*) and much larger than that of most other NCSCs (*20*). Since the above Pt-containing compounds are believed to show mixed pairing due to their large ASOC-related band splitting, also NbRuSi and TaRuSi may exhibit a significant singlet-triplet mixing. As for TiRuSi, it shows a smaller *E*_ASOC_ than NbRuSi, while HfRuSi shows a comparable *E*_ASOC_ to TaRuSi (see fig. S8). Since Ta (Hf) has a larger atomic number than Nb or Ti (and, hence, a larger SOC), it is expected that TaRuSi and HfRuSi exhibit a much larger *E*_ASOC_, in particular, considering that the *T*-metal’s *d* orbitals contribute as much as Ru-*4d* orbitals to the DOS at the Fermi energy.

**Fig. 5.**
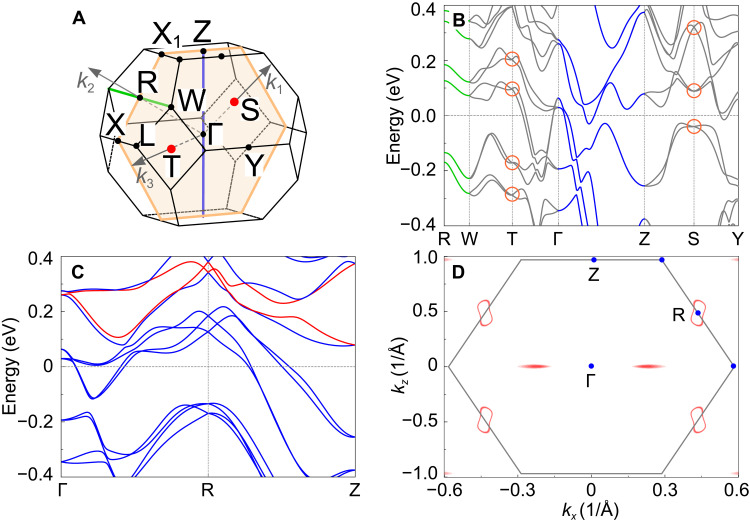
Kramers and hourglass fermions. (**A**) The first Brillouin zone of *T*RuSi materials. (**B**) Illustration of KWPs and Kramers nodal lines for TaRuSi. The KWP are marked by orange circles, while the KNL are presented by blue (along Γ − Z) or green lines (along R-W), respectively. The KWP and KNL are also depicted in the Brillouin zone in (A). (**C**) Illustration of hourglass-shaped dispersion of TaRuSi along the Γ − R − Z lines. (**D**) The shape of Weyl loops obtained from Wannier tight-binding (TB) Hamiltonian by fitting the DFT band structure (see fig. S11 for a comparison). Colors evidence the evolution of the gap between the two bands shown in red in (C). The analogous results for TiRuSi, NbRuSi, and HfRuSi can be found in figs. S8 and S10.

According to our electronic band structure calculations (see Figs. 4 and 5 and figs. S8 to S10), all members of the *T*RuSi family are symmetry-enforced semimetals, as confirmed also by the database of topological materials (*4*–*7*, *57*, *58*). In the presence of SOC, the *T*RuSi family of materials with a nonsymmorphic space group *Ima*2 (no. 46) hosts Kramers Weyl points (KWPs) at the high-symmetry points and Kramers nodal lines along the high-symmetry lines. In Fig. 5B and in figs. S8 and S10, these features are marked by orange circles (KWP) and green/blue lines (KNL). The high-symmetry points at *S* and *T* are TRS invariant. Hence, the respective energies exhibit a twofold Kramers degeneracy protected by TRS. At the same time, because of the lack of inversion symmetry, these points cannot achieve the fourfold degeneracy of Dirac points, and hence, they are Weyl points. As for the high-symmetry lines along the Γ-Z and R-W directions, the bands form a two-dimensional (2D) representation, thus, twofold degenerate, indicating the occurrence of Kramers nodal lines in *T*RuSi. As a consequence, *T*RuSi materials can be classified as Kramers nodal-line semimetals (KNLS). To the best of our knowledge, the *T*RuSi family represents the first example of KNLS to display unconventional SC with spontaneously broken TRS at *T*_c_.

### 3D bulk hourglass fermions

Now, we analyze in detail the noninteracting electronic states of the *T*RuSi materials. Because of their nonsymmorphic space group symmetry, *T*RuSi exhibit 3D bulk hourglass-type fermions, characterized by an hourglass cone with five doubly degenerate points (*15*, *17*). The nonsymmorphic space group *Ima*2 of *T*RuSi contains the glide mirror reflection generator *M_y_* = {*m*_010_∣1/2,0,0}

(x,y,z)→(x+1/2,−y,z) (1)

Here, the *k_y_* = 0- and π-planes are *M_y_*-invariant planes, where all the states along the Γ-R-Z line carry the *M_y_* index ±*ie*^−*ik_x_*/2^ and give rise to the 3D bulk hourglass fermions, protected by the *M_y_* operator (*59*, *60*). In this case, the high-symmetry point **k**-vectors are **k**_Γ_ = (0,0,0), **k***_R_* = (0,1/2,0), and **k***_Z_* = (1/2,1/2, − 1/2). Therefore, the *M_y_* index is ±1 at the *R* point and ±*i* at Γ and *Z*. In agreement with the Kramers theorem, each state is twofold degenerate, i.e., pairs of doubly degenerate states exhibit identical energies but carry opposite *M_y_* indexes. In the presence of a strong SOC, these doubly degenerate states split along the *R* → Γ or *R* → *Z* directions. Despite this SOC-induced splitting of bands with different *M_y_* indexes (see Fig. 5, C and D for TaRuSi and fig. S10 for NbRuSi), a residual degeneracy remains, which gives rise to the noninteracting hourglass fermions in *T*RuSi. To date, hourglass fermions were experimentally observed only in very few materials, as e.g., the KHgSb and Nb_3_(Si,Ge)Te_6_ topological insulators (*18*, *61*). Here, we find that also *T*RuSi belong to this restricted class of materials, where KWPs and hourglass fermions exist and can be easily shifted close to *E*_F_, either by electron doping or by chemical substitution (see figs. S12 and S13). Besides exhibiting nontrivial electronic bands, *T*RuSi also show unconventional SC with broken TRS in the superconducting state, thus representing a very rare class to combine such rather unusual electronic properties. Since the TRS is preserved in the normal state (owing to the nonmagnetic nature of *T*RuSi), by performing, e.g., ARPES measurements in both the normal and superconducting states of *T*RuSi family, one can investigate the effects of TRS breaking on these exotic electronic states and the occurrence of topological phase transitions at *T*_c_.

## DISCUSSION

In most known NCSCs, the ASOC allows, in principle, the occurrence of admixtures of spin-singlet and spin-triplet superconducting pairs, whose degree of mixing is related to the strength of ASOC and to other microscopic parameters (*19*–*21*). This sets the scene for a variety of remarkable properties. In particular, broken TRS has been shown to occur in the superconducting state of several NCSCs. To date, only a handful of weakly correlated NCSC families have been shown to exhibit TRS breaking in the superconducting state. These include LaNiC_2_ (*22*), La_7_(Rh,Ir)_3_ (*23*, *62*), Zr_3_Ir (*27*), Re*T* (*24*–*26*), CaPtAs (*29*), and, very recently, LaPt_3_P (*30*) and La*T*(Si,Ge) (*28*). Among the TRS-breaking superconductors, CaPtAs and LaPt_3_P represent a rare case, where broken TRS and nodal SC were observed to occur simultaneously below *T*_c_ (*29*, *30*). In most other cases, the SC behavior resembles that of *s*-wave superconductors, characterized by a fully opened superconducting gap and the absence of mixed pairing. Among these, only a few are known to belong to the class of topological semimetals (*30*, *63*, *64*). Here, we could show that *T*RuSi materials represent a unique family of NCSCs that not only break TRS in their superconducting state but are also topological semimetals that host KNL fermions, as well as Kramers Weyl and hourglass fermions near the Fermi energy.

Now, we discuss the relevant TRS-breaking pairing symmetry for NbRuSi and TaRuSi superconductors. A possible model should account for the following experimental aspects: (i) a fully gapped superconducting state (see Fig. 3), (ii) spontaneously broken TRS (see Fig. 2), (iii) only one superconducting transition temperature that is also the onset temperature of TRS breaking (see Figs. 1 and 3), and (iv) a large asymmetric SOC (see Fig. 4). Both NbRuSi and TaRuSi crystallize in an orthorhombic structure with a nonsymmorphic space group. Here, in view of the complexity of the electronic band structure, we focus on the Fermi surfaces around the high-symmetry Γ point to discuss the superconducting states in the weak-coupling limit. We consider a single-band model with a Rashba-type SOC, compatible with the symmetry constraints of the *C*_2*v*_ point group that admits only 1D irreducible pairing symmetries (see note S1). For instance, as summarized in Table 1, the spin-singlet pairings with $\left\{1,k_{x}^{2},k_{y}^{2},k_{z}^{2}\right\}$ belong to the *A*_1_ irreducible representation (irrep), while the spin-triplet pairings represented by the spin **d**-vector (*d*_1_*k_x_*, *d*_2_*k_y_*, *d*_3_*k_z_*) belong to the *A*_2_ irrep.

**Table 1.** Classification of pairing symmetries. The character table of the C_2v_ point group and the corresponding classification of pairing symmetries for NbRuSi and TaRuSi superconductors. The four 1D irreducible representations are labeled *A*_1_, *A*_2_, *B*_1_, and *B*_2_. For each irrep, the basis functions in both orbital space and spin subspace are listed. All the spin-singlet ψ_s_(*k*) and spin-triplet pairings $d\cdot \vec{\sigma }$ are also classified. To preserve TRS, the pairing strength *d*_1,2,3_ is set to be real; otherwise, the TRS would be spontaneously broken in case of a nonunitary spin-triplet pairing (*88*). A mixing between singlet and triplet pairings that belong to different irreps with a relative phase of ±π/2 (e.g., A_1_ + *i*A_2_) also spontaneously breaks the TRS but remains a unitary pairing.

**C_2v_**	***E***	**C_2z_**	***m_y_***	***m_x_***	**Functions**	**Spin matrix $\vec{\sigma }$**	Spin-singlet ψ_s_(***k***)	Spin-triplet **d**(**k**) vector
*A*_1_	1	1	1	1	*z*, *x*^2^, *y*^2^, *z*^2^	σ_0_	$1,k_{x}^{2},k_{y}^{2},k_{z}^{2}$	(*d*_1_*k_y_*, *d*_2_*k_x_*,0)
*A*_2_	1	1	−1	−1	*xy*	σ*_z_*	*k_x_k_y_*	(*d*_1_*k_x_*, *d*_2_*k_y_*, *d*_3_*k_z_*)
*B*_1_	1	−1	1	−1	*x*, *xz*	σ*_y_*	*k_x_k_z_*	(0, *d*_2_*k_z_*, *d*_3_*k_y_*)
*B*_2_	1	−1	−1	1	*y*, *yz*	σ*_x_*	*k_y_k_z_*	(*d*_1_*k_z_*,0, *d*_3_*k_x_*)

Several theoretical models with mixed singlet-triplet pairings have been put forward to explain the TRS-breaking effects in *s*-wave–like superconductors. In Table 2, three possible singlet-triplet TRS-breaking pairings are summarized, together with the types of induced magnetism, the symmetry reduction, as well as the condition to exist in real materials. The first pairing scenario is the internally antisymmetric nonunitary triplet (INT) pairing, which features a uniform pairing between electrons having the same spin but belongs to two different bands (no. 1 in Table 2). It can generally account for the TRS breaking in the presence of a nodeless SC. Nevertheless, the INT pairing can only exist in case of a weak SOC, because the complex spin-triplet **d**-vector is generally suppressed by large SOC. Such INT pairing was originally proposed to explain the TRS breaking and nodeless SC in LaNiGa_2_ (*65*, *66*) and LaNiC_2_ (*22*, *67*), both characterized by a relatively weak SOC. Recently, this model was applied also to La*T*(Si,Ge) and (Nb,Ta)OsSi superconductors (*28*, *68*). In this scenario, microscopic supercurrent loops with a uniform onsite and intra-orbital singlet pairing form spontaneously within the unit cell. These supercurrents may give rise to tiny static magnetic fields, thus providing a possible understanding of the broken TRS in Re*T* NCSCs (*69*). However, this scenario may not apply to the *T*RuSi superconductors due to their large asymmetric SOC (see note S1).

**Table 2.** Summary of pairing symmetries. Summary of the TRS-breaking singlet-triplet mixed pairings, including the type of magnetism (spin or orbital), symmetry reduction, condition to exist in a material, and the relevance to *T*RuSi superconductors. It is noted that the $A_{1}^{s}+e^{i\theta }A_{1}^{p}$ pairing does not break any crystal symmetries, and we label it as symmetric (*s* + *ip*) pairing. By contrast, the $A_{1}^{s}+e^{i\theta }A_{2}^{p}$ pairing breaks the crystal symmetry from C_2v_ to C_2_, which was labeled as nonsymmetric (*s* + *ip*) pairing.

**Scenario**	**TRS-breaking pairing**	**Irreps**	**Magnetism**	**Symmetry**	**Condition**	***T*RuSi relevance**
No. 1	Singlet-triplet nonunitary	$A_{1}^{s}+A_{1}^{p}$	Spin	C_2v_ → C_2v_	Weak SOC	×
No. 2	Singlet-triplet unitary	$A_{1}^{s}+iA_{2}^{p}$	Spin	C_2v_ → C_2_	Anisotropic SOC	√
No. 3	Singlet-triplet unitary/nonunitary	$A_{1}^{s}+e^{i\theta }A_{1}^{p}$	Orbital	C_2v_ → C_2v_	Impurities	√

Hence, we need to consider alternative models based on unitary pairings. One of these is the second pairing scenario (no. 2 in Table 2), where also the key role of SOC is naturally taken into account (*70*). To be explicit, because of the absence of an inversion center, the mixed singlet-triplet pairing states are generally given by $\Delta (k)=[\Delta_{s}\psi (k)+\Delta_{t}(d(k)\cdot \vec{\sigma })](i\sigma_{y})$, where Δ_s_ and Δ_t_ represent the pairing strength in spin-singlet and spin-triplet channels, respectively. According to the μSR results, NbRuSi and TaRuSi exhibit a fully gapped superconducting ground state with spontaneously broken TRS. This outcome rules out all the unitary *B*_1_ ⊗ *B*_2_ pairings with gap nodes. Therefore, a possible unitary pairing, consistent with the experimentally observed broken TRS, is *A*_1_ + *iA*_2_ (*70*). The latter, named nonsymmetric (*s* + *ip*) pairing, breaks the mirror symmetry, thus reducing the original C_2v_ point-group symmetry down to C_2_. In the weak-coupling limit, the SOC also reduces the *A*_2_ irrep’s spin-triplet component Δ_t_, since the **d**(**k**) vector is not parallel with the SOC vector (*71*, *72*). Nonetheless, the nonsymmetric (*s* + *ip*) pairing can still survive (*70*). In the presence of SOC, this pairing can generate an internal spin magnetization (see details in note S1), whose magnetic field is readily detected by the ZF-μSR technique. This magnetization lowers the free energy by $\Delta E∼-\alpha_{R}^{2}\Delta_{s}^{2}\Delta_{t}^{2}$, where α_R_ is the strength of SOC. Once Δ*E* overcomes the energy loss caused by the SOC-induced reduction of Δ_t_, the nonsymmetric (*s* + *ip*) pairing can be stabilized. Furthermore, the effect of anisotropic SOC on suppressing the *A*_2_ irrep spin-triplet pairing channel is rather weak (see details in note S2 and fig. S14).

On the basis of the above arguments, we analyzed the superfluid density of NbRuSi and TaRuSi by using this nonsymmetric (*s* + *ip*) pairing (see Fig. 3). The band structure calculations reveal that the Rashba-type SOC is highly anisotropic on the Fermi surfaces around the Γ point. Therefore, we also have to consider an anisotropic spin-triplet **d** vector with *d*_1_ = 1 but *d*_2_ = *d*_3_ = 0 that may be stabilized (see note S2). In case of a weak SOC, the nonsymmetric (*s* + *ip*) pairing gives a gap function $\Delta ∼\sqrt{\Delta_{s}^{2}\psi_{s}^{2}+\Delta_{t}^{2}}$, which is similar to an *s*-wave gap function, if ψ_s_ ∼ 1 is assumed. By contrast, in the strong SOC limit, when ignoring the interband pairing, the intraband pairing function is given by $\Delta ∼\sqrt{\Delta_{s}^{2}+\Delta_{t}^{2}{\left({\text{sin}}^{2}\text{θsin}\;2\phi \right)}^{2}}$. In this case, gap nodes will appear only if Δ_s_ ≪ Δ_t_. While in the Δ_s_ ≳ Δ_t_ case, the gap is fully oppened, reflecting the strong mixing of spin-singlet with spin-triplet pairing. Both gap functions can describe the superfluid density very well (see details in Fig. 3, E and F). Therefore, we may conclude that the nonsymmetric (*s* + *ip*) pairing is especially relevant for the NbRuSi and TaRuSi superconductors.

In addition, also a symmetric (*s* + *ip*) pairing, i.e., a third pairing scenario (no. 3 in Table 2) is possible. In this case, both the spin-singlet and spin-triplet channels belong to the same *A*_1_ irrep. We also analyzed the superfluid density by using the superconducting gap function $\Delta (\theta ,\phi )\approx \sqrt{1+{\text{sin}}^{2}\theta }$ by assuming Δ_s_ ≃ Δ_t_ of the symmetric (*s* + *ip*) pairing, which is also in good agreement with the experimental data (see fig. S7 and table S1). Such pairing implies a fully gapped SC, but it does not generate spin or orbital magnetism. For a clean system, the symmetric (*s* + *ip*) pairing also fails to explain the broken TRS in the superconducting state of NbRuSi and TaRuSi. However, the presence of impurities or lattice defects can locally break the superconducting states and induce supercurrent loops ${\vec{J}}_{s}$ that, in turn, generate a local magnetic field via the Ampère’s circuital law ∇ × $\vec{B}$ = 4π/c${\vec{J}}_{s}$. However, distinguishing a symmetric (*s* + *ip*) pairing (in “dirty” systems) from a nonsymmetric one (in clean systems) is not an easy task, and it requires further investigations, e.g., μSR studies on high-quality NbRuSi and TaRuSi single crystals.

Last, besides the *T*RuSi family studied here, the isostructural TaReSi also becomes a superconductor below *T*_c_ = 5.3 K (*43*). By substituting the 4*d*-Ru with 5*d*-Re, the ASOC strength increases progressively from TiRuSi to TaReSi. Therefore, it would be interesting to search for possible TRS breaking and, hence, unconventional or topological SC in TaReSi. In addition, considering that both Kramers and hourglass fermions are closer to the Fermi level in TiRuSi and HfRuSi (see fig. S8), Ti_1−*x*_(Nb,Ta)*_x_*RuSi or TaRu_1−*x*_Re*_x_*Si lends themselves as ideal systems for investigating the relationship between ASOC and unconventional SC with TRS breaking, as well as their interplay with the exotic Kramers and hourglass fermions. On the other hand, we also calculated the surface states of *T*RuSi compounds and found clear spin-polarized Fermi arcs that cross the Fermi level (see the TaRuSi example in fig. S15). In centrosymmetric TRS-broken Weyl semimetals, the Fermi arcs cannot host a momentum counterpart. Conversely, in time-reversal symmetric Weyl semimetals, Fermi arcs can host spin-polarized surface states with momentum counterparts, which could be driven into SC by proximity effect. Similar to the surface topological SC, formed on the surface of topological insulators (*73*), the surface Fermi arcs in *T*RuSi materials can give rise to a novel 2D surface SC (*74*), a new type of topological order, to be confirmed by future experimental studies.

In conclusion, we have found a unique family of NCSCs, namely, *T*RuSi (with *T* = Nb and Ta), which shows nontrivial band topology and breaks TRS below the superconducting transition. Theoretically, we propose a unitary (*s* + *ip*) mixed pairing to account for the experimental observations of a fully gapped superconducting state with broken TRS in the presence of a strong SOC. Band structure calculations reveal that *T*RuSi (*T* = Ti, Nb, Hf, and Ta) are 3D KNLS, which also feature hourglass fermions protected by their nonsymmorphic space group symmetry. Our results demonstrate that *T*RuSi materials represent an extremely rare case of topological semimetals, which show unconventional SC at low temperatures. Hence, they epitomize the ideal system for investigating the rich interplay between the exotic electronic states of KNL fermions, hourglass fermions, and unconventional SC. Considering the nontrivial band structure and surface states near the Fermi level and the intrinsic unconventional SC, the *T*RuSi family may provide a very promising platform for realizing topological SC, with potential applications in future quantum computation.

## MATERIALS AND METHODS

### Materials synthesis

Polycrystalline *T*RuSi (*T* = Ti, Nb, Hf, and Ta) samples were prepared by arc melting stoichiometric pieces of Ti (99.98%), Nb (99.95%), Hf (99.9%), or Ta (99.98%) slugs, Ru (99.9%) powders, and Si chunks (99.9999%) in a high-purity argon atmosphere. Except for Ru (ChemPUR), all materials were provided by Alfa Aesar. To improve sample homogeneity, the ingots were flipped and remelted more than six times. The resulting samples were annealed at 800^∘^C for 2 weeks and lastly quenched in water.

### Sample characterization

The crystal structure of *T*RuSi samples was checked via powder XRD at room temperature using a Bruker D8 diffractometer with Cu K_α_ radiation. This confirmed the orthorhombic noncentrosymmetric structure of *T*RuSi (*Ima*2, no. 46). The electrical resistivity, heat capacity, and magnetization measurements were performed on a Quantum Design physical property measurement system and a magnetic property measurement system. The lower critical fields *H*_c1_, essential for performing TF-μSR in type II superconductors, were determined by field-dependent magnetization measurements at various temperatures up to *T*_c_.

### μSR experiments

The μSR measurements were carried out at the multipurpose surface muon spectrometer (Dolly) of the Swiss muon source (SμS) at the Paul Scherrer Institut, Villigen, Switzerland. In this study, we performed three kinds of experiments: TF-, ZF-, and LF-μSR measurements. As to the former, it allowed us to determine the temperature evolution of the superfluid density. As to the latter two, we aimed at searching for a possible breaking of TRS in the superconducting state of NbRuSi and TaRuSi. In the TF-μSR measurements, the applied magnetic field was perpendicular to the muon-spin direction, while in the LF-μSR measurements, the magnetic field was parallel to the muon-spin direction. In both cases, the samples were cooled in an applied magnetic field down to the base temperature (∼0.3 K), and then the μSR spectra were collected upon heating. To exclude the possibility of stray magnetic fields during the ZF-μSR measurements, all the magnets were preliminarily degaussed, and we made use of an active field-nulling facility (*75*).

### Analysis of the μSR spectra

All the μSR data were analyzed by means of the musrfit software package (*76*). In the TF-μSR case, to properly describe the asymmetric field distribution [see the fast Fourier transform (FFT) spectra in fig. S6], the μSR spectra were modeled by

$$A(t)=\sum_{i=1}^{n}A_{i}\text{cos}(\gamma_{\mu }B_{i}t+\phi )e-\sigma_{i}^{2}t^{2}/2+A_{\text{bg}}\text{cos}(\gamma_{\mu }B_{\text{bg}}t+\phi )$$ (2)

Here, *A_i_*, *A*_bg_ and *B_i_*, *B*_bg_ are the initial muon-spin asymmetries and local fields sensed by the implanted muons in the sample and sample holder (here, a copper plate exhibiting zero muon-spin depolarization), γ_μ_/2π = 135.53 MHz/T is the muon gyromagnetic ratio, ϕ is a shared initial phase, and σ*_i_* is the Gaussian relaxation rate of the *i*th component. According to the FFT of TF-μSR spectra in the superconducting state, a single oscillation fails to reproduce the additional field distribution broadening due to FLL in the mixed state. On the other hand, two oscillations (i.e., *n* = 2) reproduce quite well the NbRuSi and TaRuSi data (see details in fig. S6).

In case of multicomponent oscillations, the first term in Eq. 2 describes the field distribution as a sum of *n* Gaussian relaxations (here, *n* = 2) (*77*)

$$P(B)=\gamma_{\mu }\sum_{i=1}^{2}\frac{A_{i}}{\sigma_{i}}\text{exp}[-\frac{\gamma_{\mu }^{2}{\left(B-B_{i}\right)}^{2}}{2\sigma_{i}^{2}}]$$ (3)

The first and second moments of the field distribution can be calculated by

$$\begin{array}{c} \langle B\rangle =\sum_{i=1}^{2}\frac{A_{i}B_{i}}{A_{\text{tot}}},\text{and}\\ \langle B^{2}\rangle =\frac{\sigma_{\text{eff}}^{2}}{\gamma_{\mu }^{2}}=\sum_{i=1}^{2}\frac{A_{i}}{A_{\text{tot}}}[\frac{\sigma_{i}^{2}}{\gamma_{\mu }^{2}}+{\left(B_{i}-\langle B\rangle \right)}^{2}] \end{array}$$ (4)

where *A*_tot_ = *A*_1_ + *A*_2_. Considering that the changes in nuclear relaxation rate σ*_n_* can be ignored compared to the contribution due to the FLL (see also ZF-μSR in Fig. 2), the superconducting contribution can be extracted using $\sigma_{\text{sc}}=\sqrt{\sigma_{\text{eff}}^{2}-\sigma_{n}^{2}}$. Then, the effective magnetic penetration depth λ_eff_ can be calculated by (*78*, *79*)

$$\frac{\sigma_{\text{sc}}^{2}(T)}{\gamma_{\mu }^{2}}=0.00371\;\frac{\Phi_{0}^{2}}{\lambda_{\text{eff}}^{4}(T)}$$ (5)

Since σ_sc_ is directly related to the effective magnetic penetration depth and, thus, to superfluid density ($\sigma_{\text{sc}}\propto 1/\lambda_{\text{eff}}^{2}∼\rho_{\text{sc}}$), the superconducting gap and its symmetry can be investigated by measuring the temperature-dependent σ_sc_.

For NbRuSi and TaRuSi, the ZF-μSR spectra can be modeled by means of a phenomenological relaxation function, consisting of a combination of Gaussian and Lorentzian Kubo-Toyabe relaxations (*49*, *50*)

$$A_{\text{ZF}}=A_{s}[\frac{1}{3}+\frac{2}{3}(1-\sigma_{\text{ZF}}^{2}t^{2}-\Lambda_{\text{ZF}}t)\;e^{\left(-\frac{\sigma_{\text{ZF}}^{2}t^{2}}{2}-\Lambda_{\text{ZF}}t\right)}]+A_{\text{bg}}$$ (6)

Here, *A*_s_ (≡ *A*_tot_) and *A*_bg_ are the same as in the TF-μSR case. The σ_ZF_ and Λ_ZF_ represent the ZF muon-spin relaxation rates of the Gaussian and Lorentzian term, respectively. This model has been frequently applied to analyze the ZF-μSR in other superconductors. Since Λ_ZF_ shows an almost temperature-independent behavior (see fig. S5), the σ_ZF_ values in Fig. 2 (C and D) were obtained by fixing Λ_ZF_ to their average values, i.e., $\Lambda_{\text{ZF}}^{\text{av}}=0.005$ and 0.010 μ*s*^−1^ for NbRuSi and TaRuSi, respectively. The σ_ZF_(*T*) values derived using a free- or a fixed-Λ_ZF_ analysis are highly consistent, thus confirming the unbiased evaluation of relaxation below *T*_c_.

### Superconducting gap symmetry

The superfluid density ρ_sc_(*T*) of NbRuSi and TaRuSi was analyzed within the London approximation, generally described by (*77*, *80*)

$$\begin{array}{c} \rho_{\text{sc}}{\left(T\right)}^{\mathrm{aa}/\mathrm{bb}/\mathrm{cc}}=\frac{\lambda_{0}^{2}}{\lambda_{\text{eff}}^{2}(T)}=1-\frac{3}{4\pi T}\int (\begin{array}{c} {\text{sin}}^{2}\theta {\text{cos}}^{2}\phi \\ {\text{sin}}^{2}\theta {\text{sin}}^{2}\phi \\ 2{\text{cos}}^{2}\theta {\text{cos}}^{2}\phi \end{array})\\ \times {\text{cosh}}^{-2}(\frac{E^{2}+\Delta_{k}^{2}}{2T})dE\text{dθd}\phi \end{array}$$ (7)

Here, *f* = (1 + *e*^*E*/*k*_B_^^*T*^)^−1^ is the Fermi function and λ_0_ is the effective magnetic penetration depth at zero temperature (*80*, *81*). Δ_k_(*T*) = Δ(*T*)*g*_k_ is an angle-dependent gap function, where Δ is the maximum gap value and *g*_k_ is the angular dependence of the gap. The temperature dependence of the gap is assumed to follow Δ(*T*) = Δ_0_ tanh {1.82[1.018(*T*_c_/*T* − 1)]^0.51^}, where Δ_0_ is the gap value at 0 K (*82*). $\rho_{\text{sc}}^{\mathrm{aa}/\mathrm{bb}/\mathrm{cc}}$ is the component of the superfluid density along *a*, *b*, and *c* axes. Since our μSR experiments were performed on NbRuSi and TaRuSi polycrystalline samples, we used the average superfluid density $\rho_{\text{sc}}=(\sqrt{\rho_{\text{sc}}^{\mathrm{aa}}\rho_{\text{sc}}^{\mathrm{bb}}}+\sqrt{\rho_{\text{sc}}^{\mathrm{aa}}\rho_{\text{sc}}^{\mathrm{cc}}}+\sqrt{\rho_{\text{sc}}^{\mathrm{bb}}\rho_{\text{sc}}^{\mathrm{cc}}})/3$ to analyze the TF-μSR data (see lines in Fig. 3, E and F) (*77*).

### Electronic band structure calculations

First-principles calculations were performed on the basis of the DFT, as implemented in the Quantum ESPRESSO package (*83*, *84*). The exchange correlation functional is treated with the generalized gradient approximation using the Perdew-Burke-Ernzerhof realization (*85*). The projector-augmented wave pseudopotentials are adopted (*86*). We considered 12 electrons for Ti (3*s*^2^4*s*^2^3*p*^6^3*d*^2^), 13 electrons for Nb (4*s*^2^5*s*^2^4*p*^6^4*d*^3^), 12 electrons for Hf (5*s*^2^6*s*^2^5*p*^6^5*d*^2^), 13 electrons for Ta (5*s*^2^6*s*^2^5*p*^6^5*d*^3^), 16 electrons for Ru (4*s*^2^5*s*^2^4*p*^6^4*d*^6^), and 4 electrons for Si (3*s*^2^3*p*^2^) as valence electrons. SOC effects were included in the calculation. The kinetic energy cutoff for the wave functions was set to 60 Ry, while for the charge density, it was fixed to 600 Ry. For the self-consistent calculations, the Brillouin zone integration was performed on Monkhorst-Pack grid mesh of 10 × 10 × 10 *k*-points. The convergence criterion was set to 10^−7^ Ry. Last, the calculations relied on the experimental lattice parameters. To calculate the band geometric quantities, a Wannier tight-binding Hamiltonian, consisting of Ta-5*d*, Nb-4*d*, Ru-4*d*, and Si-3*p* orbitals, was constructed using the Wannier90 package (*87*).

## Supplementary Materials

### This PDF file includes:

Figs. S1 to S15 Table S1 Note S1 and S2
