# Supplementary material for: Unconventional superconductivity in topological Kramers nodal-line semimetals
Source: Sci Adv. 2022 Oct 28;8(43):eabq6589. doi: 10.1126/sciadv.abq6589 (PMC9616505; doi:10.1126/sciadv.abq6589)
Supplement: Supplementary file 1 — Figs. S1 to S15 Table S1 Note S1 and S2 [file sciadv.abq6589_sm.pdf]

Science Advances
AAAS

Supplementary Materials for

Unconventional superconductivity in topological Kramers nodal-line semimetals

Tian Shang *et al.*

Corresponding author: Tian Shang, Toni Shiroka, tshang@phy.ecnu.edu.cn; Tian Shang, Toni Shiroka, tshiroka@phys.ethz.ch

Sci. Adv. **8**, eabq6589 (2022)
DOI: 10.1126/sciadv.abq6589

This PDF file includes:

Figs. S1 to S15
Table S1
Note S1 and S2

Supplementary Materials to

"Unconventional superconductivity in topological Kramers nodal-line semimetals"

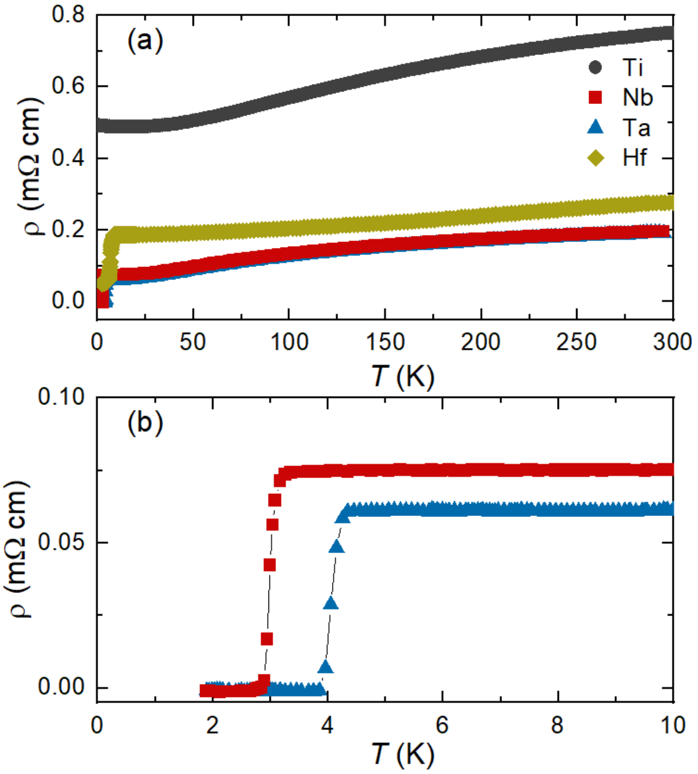

Figure S1 (a) Temperature-dependent electrical resistivity of *T*RuSi (*T* = Ti, Nb, Ta, and Hf) from 300 K down to 2 K in the absence of a magnetic field. (b) The enlarged plot shows the resistivity of NbRuSi and TaRuSi below 10 K. Note that, in HfRuSi, the transition at 8.8 K might be due to a minority superconducting phase (i.e., HfRu), as confirmed by the magnetic susceptibility data in Figure S2 below.

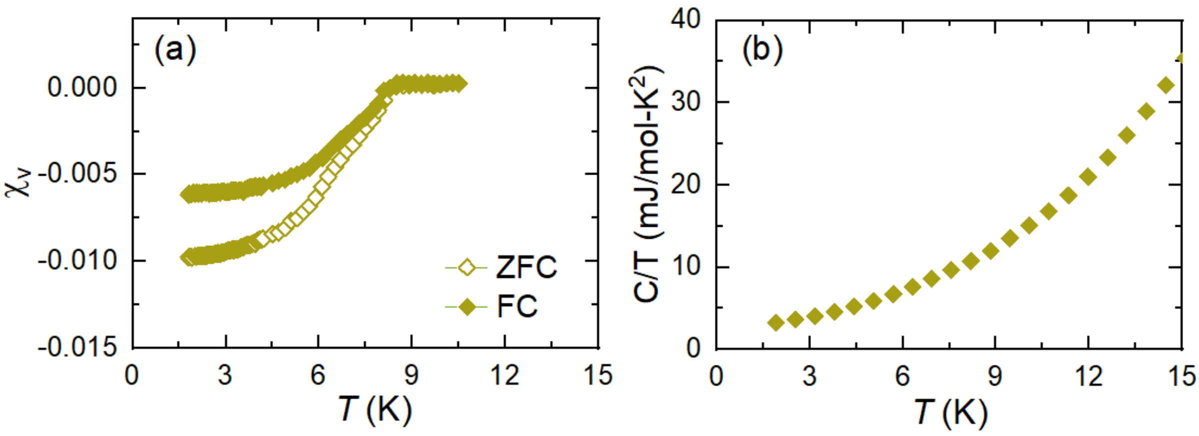

Figure S2 Temperature-dependent magnetic susceptibility (a) and specific heat (b) of HfRuSi. The susceptibility was measured in an applied field of 1 mT using the ZFC and FC protocols. Note that the superconducting volume fraction is only a marginal 2%, as confirmed by the absence of any anomaly in the specific-heat data at 8.8 K.

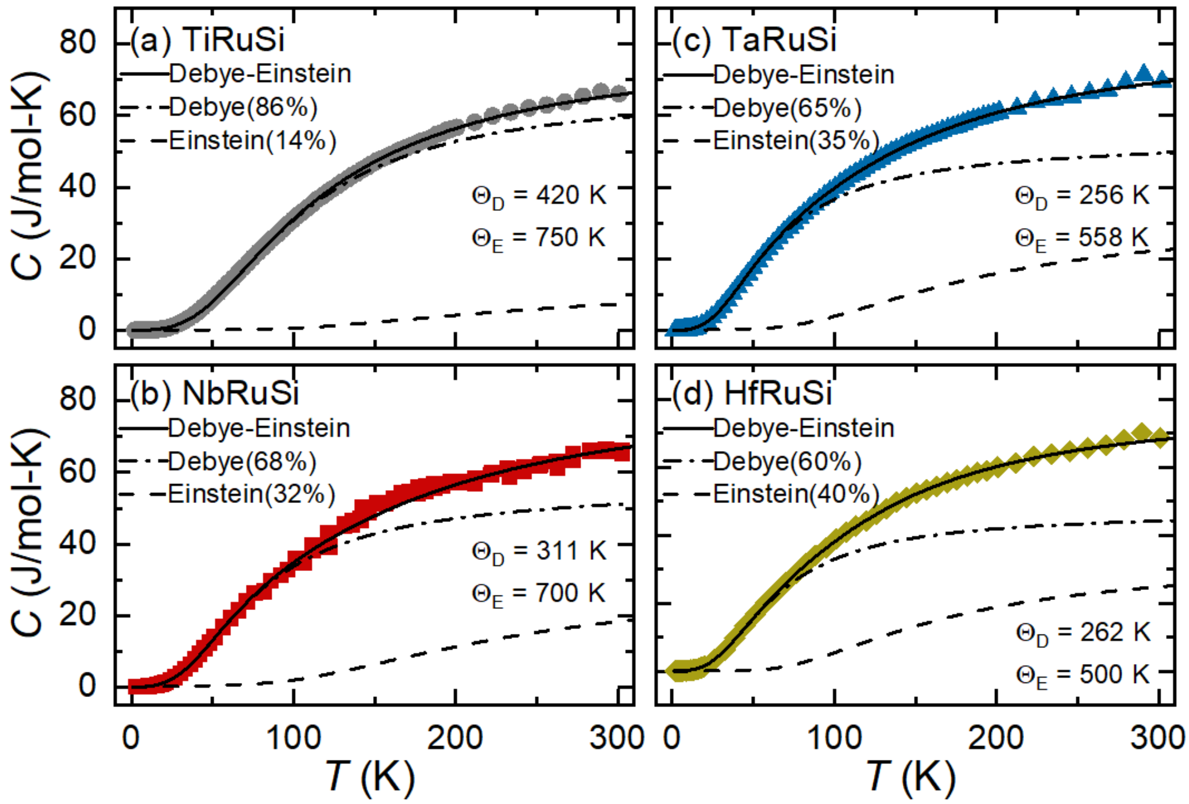

Figure S3 Temperature-dependent heat capacity of *T*RuSi (*T* = Ti, Nb, Ta, and Hf), measured in zero field from 2 to 300 K. The solid lines represent fits to a combined Debye- and Einstein model, with the dash-dotted- and dashed lines referring to the two components. The estimated Debye- and Einstein temperatures are listed in the respective panels. The resulting electronic specific-heat coefficients are $\gamma_n = 3.1$, 9.4, 8.4, and 2.9 mJ/mol-K^2 for TiRuSi, NbRuSi, TaRuSi and HfRuSi, respectively.

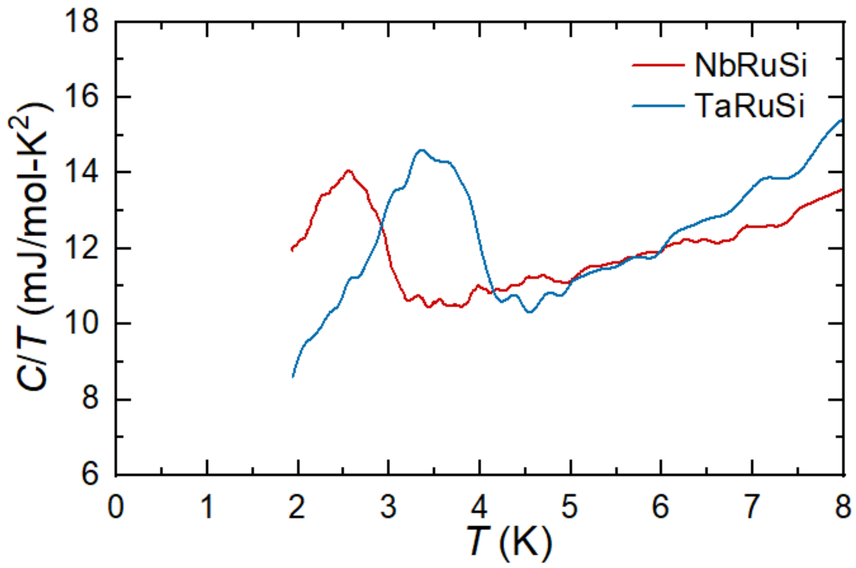

Figure S4 Temperature-dependent specific heat *C*(*T*)/*T* of NbRuSi and TaRuSi at low temperature. Note the clear jump at the respective superconducting transitions.

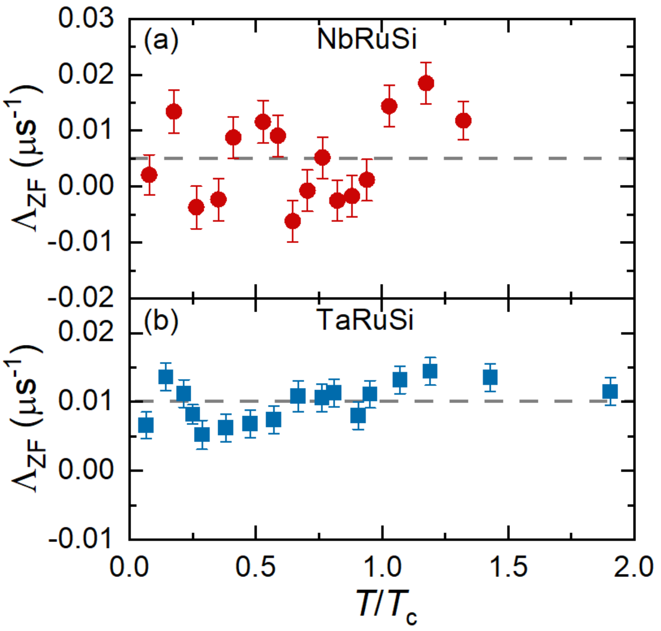

Figure S5 Nuclear-moment-related Lorentzian relaxation rates Λ_{ZF} derived from the ZF-μSR data vs. reduced temperature T/T_c for NbRuSi (a) and TaRuSi (b), respectively. In either case, $\Lambda_{ZF}(T)$ does not show anomalies across T_c, although $\sigma_{ZF}(T)$ (see main text) shows a weak but clear increase in the superconducting state. The dashed lines mark the average value of Λ_{ZF}.

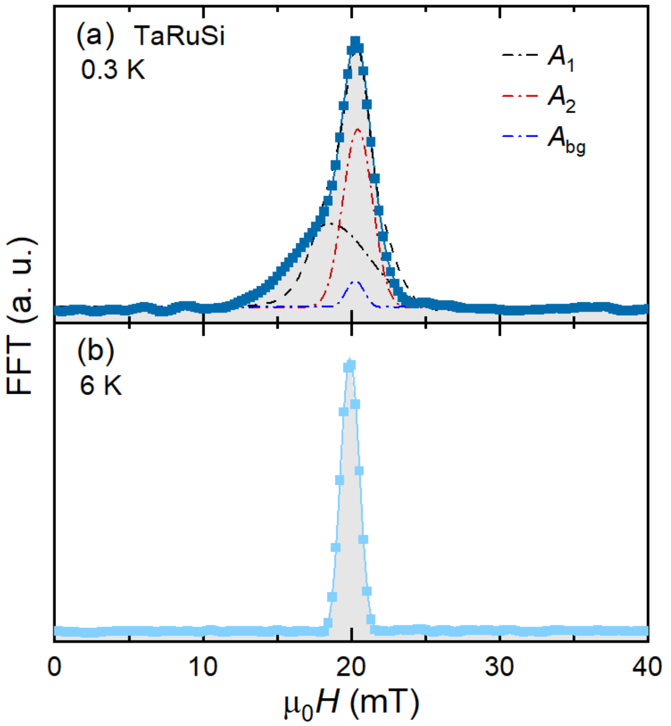

Figure S6 Real part of the Fast Fourier transforms of the TF-μSR data of TaRuSi, collected at 0.3 K (a) and 6 K (b), and shown in Figure 3(b) of the main text. Solid lines are fits to Eq. (1) in the main text using two oscillations, here shown also separately as dash-dotted lines, together with a background contribution; while the black dashed line represents a fit to Eq. (1) with a single oscillation, clearly in poor agreement with the experimental data. NbRuSi exhibits similar features.

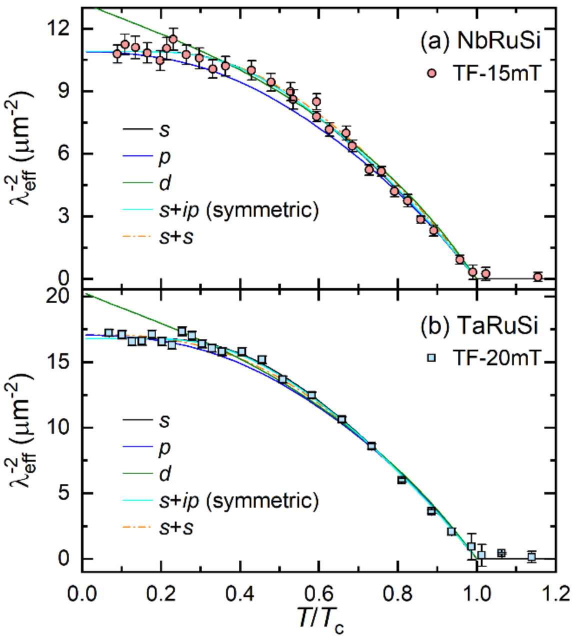

Figure S7 Superfluid density vs. reduced temperature T/T_c for NbRuSi (a) and TaRuSi (b). The solid lines are fits to the single-gap *s*-, *p*-, *d*-, and symmetric (*s*+*ip*)-wave models, while the dash-dotted lines represent fits to the (*s*+*s*)-wave model with two gaps. Here the *s*-wave model is identical to the (*s*+*ip*) model without considering SOC in the main text. It is noted that the (*s*+*ip*) model in the main text is non-symmetric.

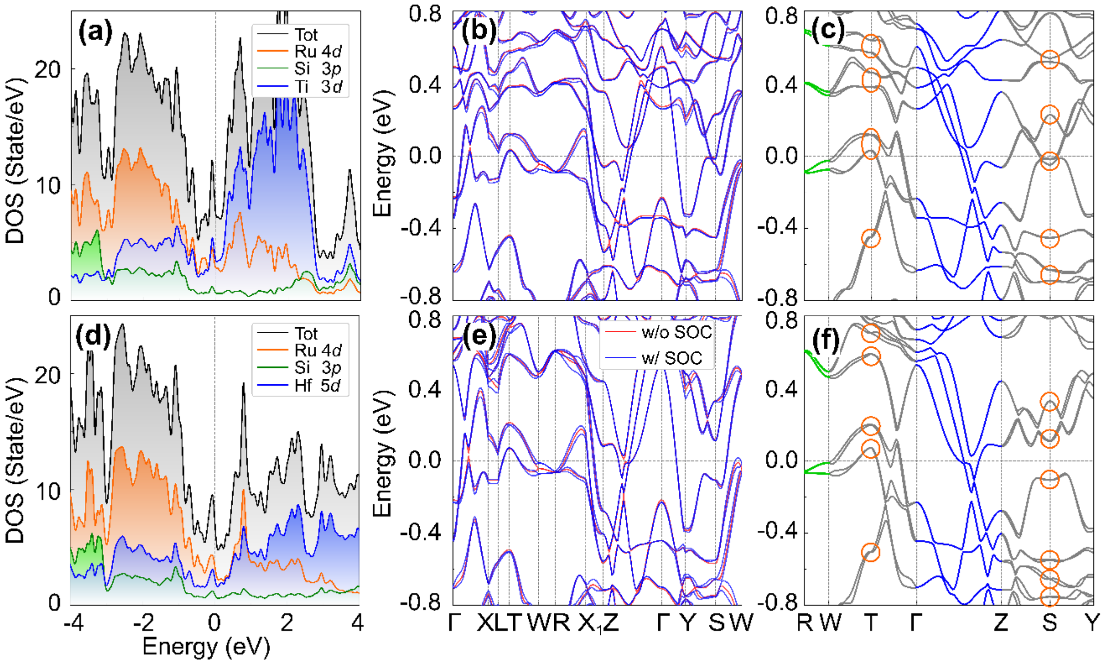

Figure S8 (a)-(c) Calculated density of states and electronic band structures for TiRuSi. (d)-(f) The analogous results for HfRuSi. The band structures in (b) and (e) were calculated by ignoring and by considering the spin-orbit coupling, here shown with red and blue lines, respectively. In (c) and (f), the Kramers Weyl points and nodal lines are marked by orange circles and are presented by blue- (along *Z*-*Γ*) or green lines (along *R*-*W*), respectively.

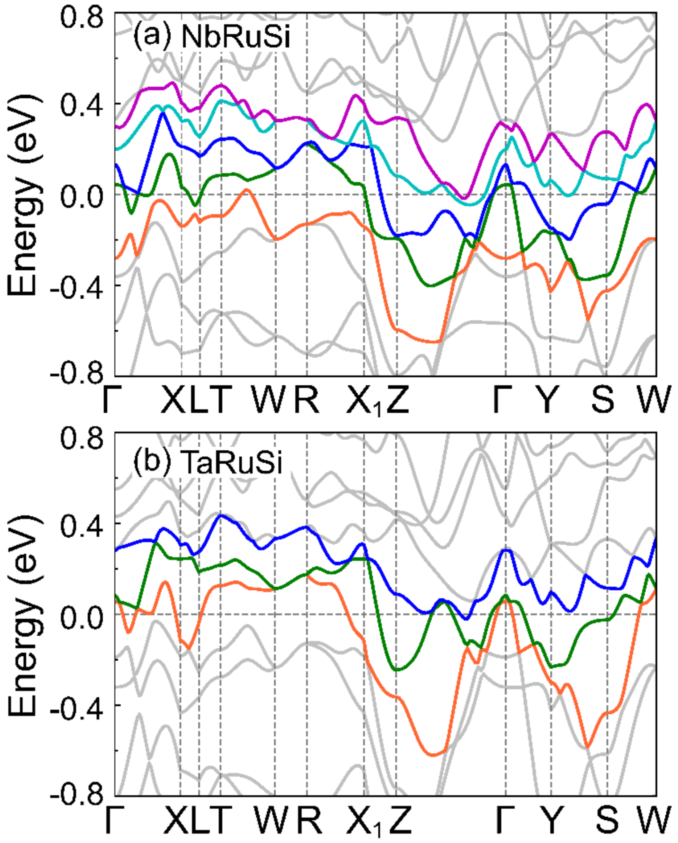

Figure S9 Electronic band structures of NbRuSi (a) and TaRuSi (b), calculated by ignoring the spin-orbit coupling. The multiple bands which cross the Fermi level are highlighted using different colors. In NbRuSi and TaRuSi, five- (top panel) and three (bottom panel) bands, respectively, are identified to cross the Fermi level.

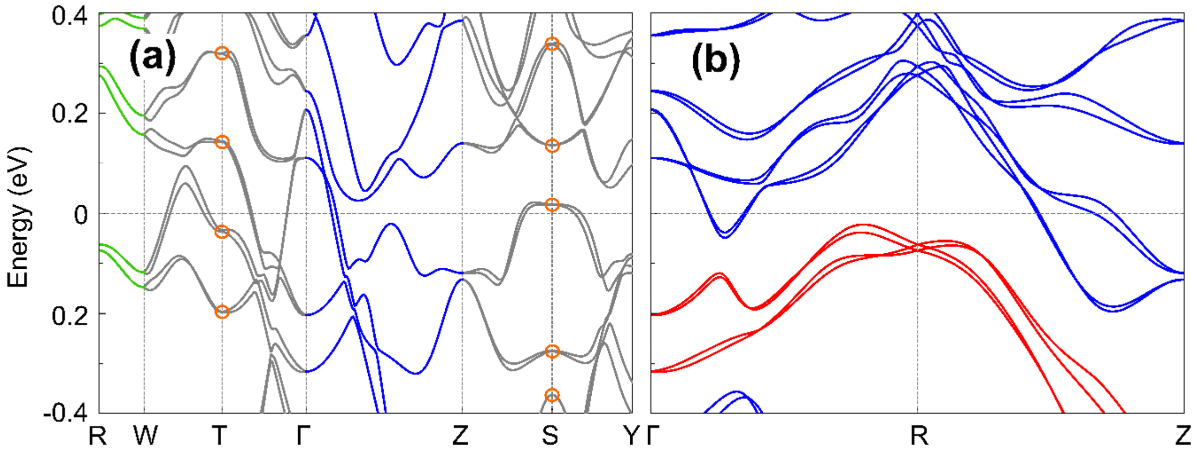

Figure S10 (a) Demonstration of Kramers Weyl points and Kramers nodal lines for NbRuSi. The KWP are marked by orange circles, while the KNL are presented by blue (along *Γ−Z*) or green lines (along *R−W*), respectively. (b) Demonstration of hourglass dispersion for NbRuSi along the *Γ-R-Z* direction.

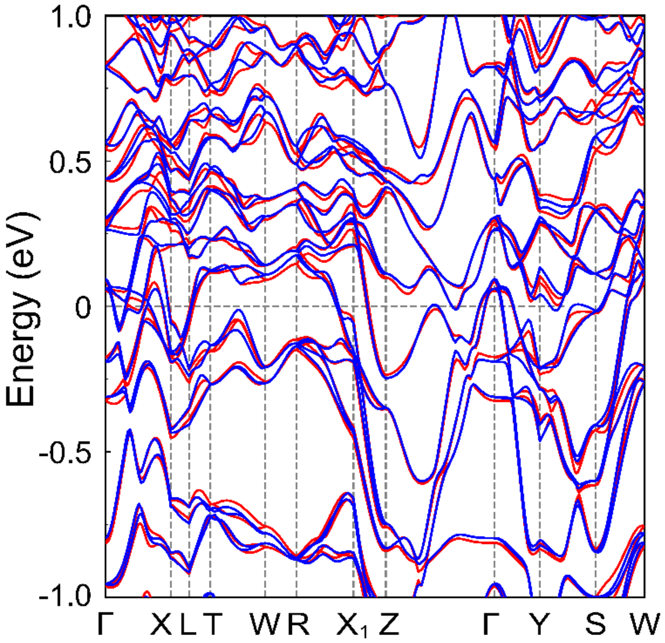

Figure S11 Comparison between the TaRuSi electronic band structures calculated using DFT- (red lines) and Wannier tight binding (blue lines) methods.

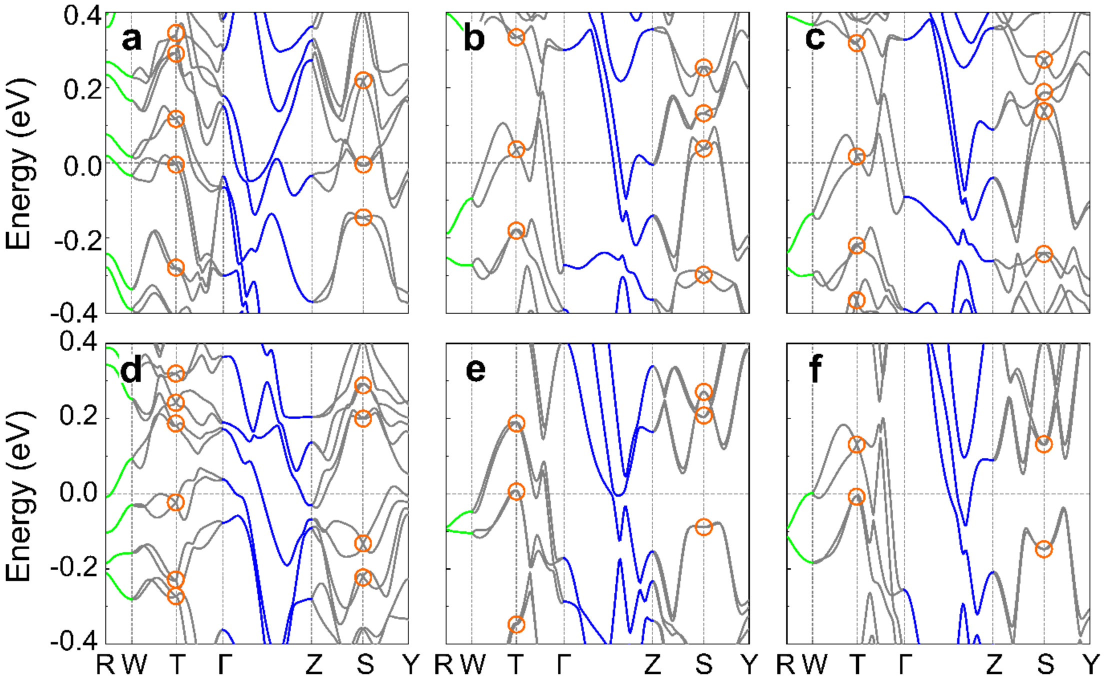

Figure S12 Illustration of Kramers Weyl points and Kramers nodal lines for (a) TaRuSi (doped with1.5 electrons per cell), (b) TiOsSi, (c) TiOsGe, (d) TiIrGe, (e) ZrRuGe, (f) HfOsSi. Similar to *T*RuSi, presented in the manuscript, all these materials adopt the same TiFeSi-type crystal structure (*Ima*2, No. 46). The KWP are marked by orange circles, while the KNL are presented by blue- (along Γ-Z) or green lines (along R-W), respectively. Clearly, the KWP and KNL can be shifted to E_F either by electron doping (e.g., deposition of potassium) or by chemical substitution. In certain cases, the Kramers Weyl points are already located at E_F [see, e.g., the T point in panels (a), (e), and (f)].

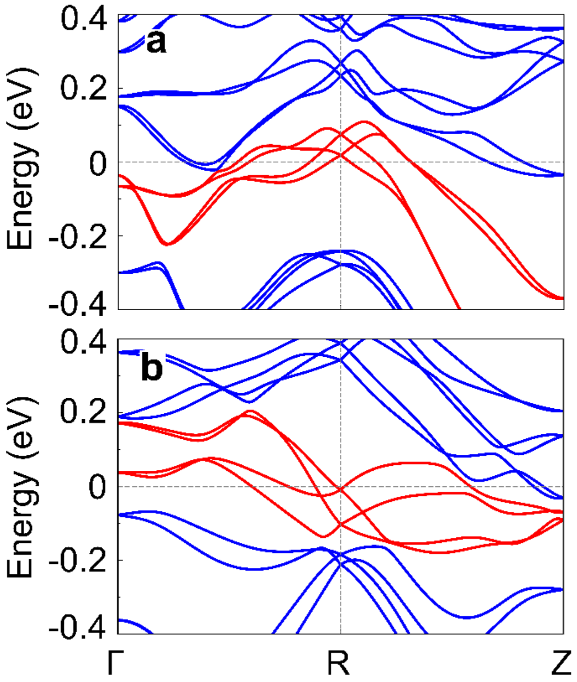

Figure S13 Illustration of hourglass-shaped dispersion of (a) TaRuSi (doped with 1.5 electrons per cell) and (b) TiIrGe along the Γ-R-Z lines. In both cases, the hourglass dispersion crosses the Fermi level E_F located at 0 eV.

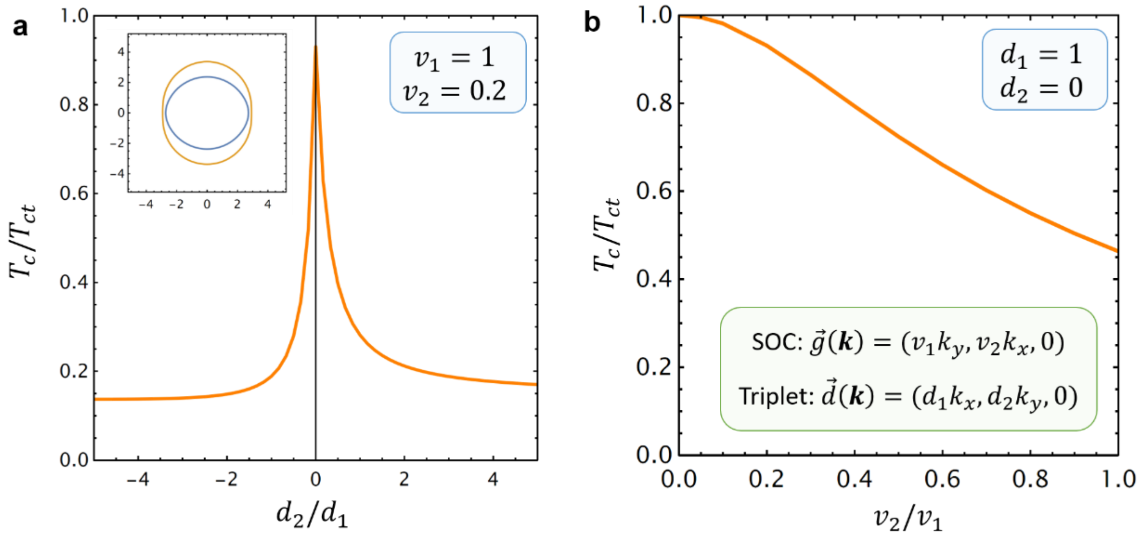

Figure S14 The effect of anisotropic SOC on the A_2 irrep spin-triplet pairing. (a) The calculated T_c/T_{ct} as a function of d_2/d_1 for $v_1 = 1$ and $v_2 = 0.2$. The inset shows the two Fermi surfaces with anisotropic SOC. (b) The calculation of T_c/T_{ct} as a function of v_2/v_1 for $d_2 = 0$. The parameters used here are: $m_z = 1, E_F = 8,$ and $\alpha_R = 1$.

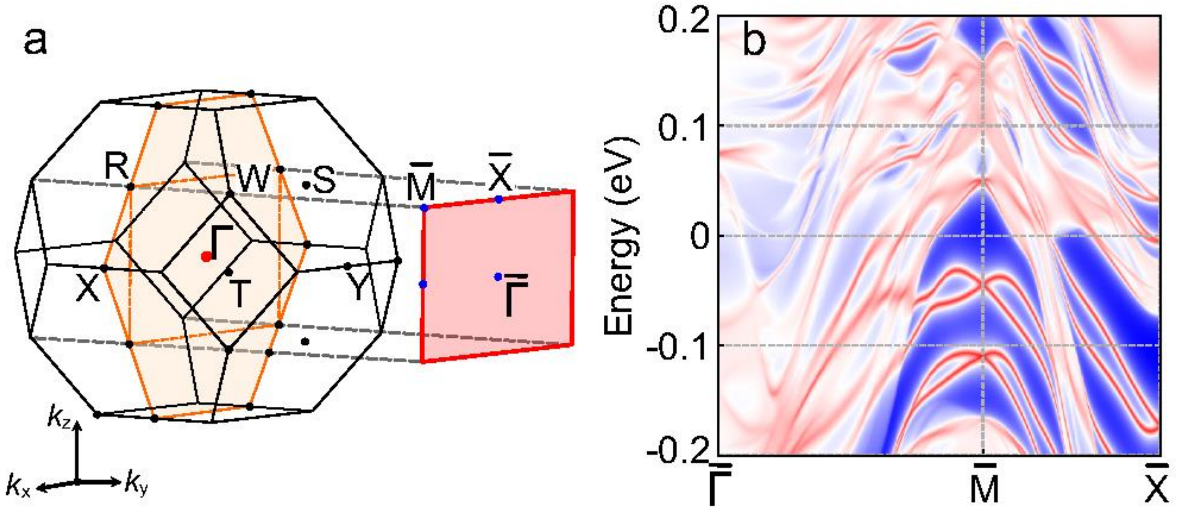

Figure S15 (a) The first Brillouin zone of bulk and (010) surface. (b) (010) surface states along the $\bar{\Gamma}-\bar{M}-\bar{X}$ path.

Table S1 Summary of the superfluid-density analysis using different models for NbRuSi and TaRuSi. In the last column, the reduced least-square deviations χ_r^2 are reported. Note that the *s*-wave model is identical to the (*s*+*ip*) models without considering SOC. For the (*s*+*s*)-wave model, the weights (w) listed in the table refer to the first *s* component. For the (*s*+*ip*)-wave models, the gap values depend strongly also on the parameters Δ_s and Δ_t (see details in the main text). Here, the gap sizes of the (*s*+*ip*) models refer to the $\Delta_t/\Delta_s \sim 1$.

		NbRuSi			TaRuSi		
Model	Gap type	λ_0 (nm)	Gap (k_BT_c)	χ_r^2	λ_0 (nm)	Gap (k_BT_c)	χ_r^2
s	nodeless	303	1.80	1.4	244	1.85	1.72
s+*ip* (SOC) (non-symmetric)	nodeless	303	1.60	1.0	244	1.60	1.59
s+*ip* (symmetric)	nodeless	303	1.40	1.0	244	1.40	1.75
p	point-node	303	2.3	3.8	242	2.3	4.0
d	line-node	275	2.5	6.7	222	2.45	14.0
s+*s* (w)	nodeless	303	1.5/2.0(0.3)	1.1	244	1.7/2.0(0.6)	2.16

Note S1

In the main text, we use a simplified $\boldsymbol{k}\cdot\boldsymbol{p}$-type Hamiltonian, given by:

$$H_0(\boldsymbol{k}) = \epsilon(\boldsymbol{k})\sigma_0 + \alpha_R\vec{g}(\boldsymbol{k})\cdot\vec{\sigma},$$

where $\epsilon(\boldsymbol{k}) = m_x k_x^2 + m_y k_y^2 + m_z\, k_z^2$, and $\vec{g}(\boldsymbol{k}) = (v_1 k_y, v_2 k_x, 0)$ is the Rashba-type spin-orbital coupling. Here, the Pauli matrix $\vec{\sigma}$ denotes the spin degree of freedom, and $m_x, m_y, m_z, \alpha_R, v_1, v_2$ are free parameters that depend on the material details. We note that higher order k^3 terms, as e.g., $k_x k_y k_z \sigma_z$, are ignored, since along the $\Gamma - Z$ direction the SOC is rather weak, and it can be ignored close to the Fermi energy (see Figs. 4B and 4E in the main text). In the case of a clean superconducting system, we find that the singlet-triplet $A_1 + iA_2$ unitary pairing is compatible with the NbRuSi and TaRuSi superconductivity. Here the A_1 irreducible representation (irrep) accounts for the spin-singlet channel (Δ_s) and the A_2 irrep represents the spin-triplet channel (Δ_t). Thus, we label this pairing symmetry $A_1^s + iA_2^p$ as non-symmetric (*s*+*ip*) pairing, because it naturally breaks the crystal symmetry, i.e., by reducing the C_{2v} point group down to the C_2 point group. However, it is well known that the non-A_1 irrep spin-triplet **d**-vector would be suppressed by the presence of SOC. Namely, the spin-triplet pairing strength Δ_t is suppressed by the SOC, which in turn increases the superconducting free energy. Nevertheless, such non-symmetric (*s*+*ip*) pairing can still be stabilized by the formation of superconductivity-induced spin magnetism.

The superconductivity-induced internal spin magnetization in the broken-TRS state can be understood based on the standard Ginzburg-Landau theory. The symmetry-allowed Ginzburg-Landau free energy for a homogeneous superconductor is given by:

$$F = \alpha_s(T)|\Delta_s|^2 + \alpha_t(T)|\Delta_t|^2 + \alpha_M\left|\vec{M}_s\right|^2 + \gamma_1(\Delta_s\Delta_t)^2 + \gamma_2 M_z \Delta_s \Delta_t + c.c.,$$

where $\Delta_s, \Delta_t, \vec{M}_s$ are the order parameters for spin-singlet pairing, spin-triplet pairing, and spin-magnetization, respectively. Here, the high-order terms are not shown. The $\alpha_s(T)$ and $\alpha_t(T)$ determine the superconducting transition temperatures and are assumed to be close in two channels due to the absence of inversion symmetry. There is no spontaneous ferromagnetic ordering in the currently studied materials, therefore, we can simply set $\alpha_M > 0$. Here, $\gamma_1 \neq 0$ gives rise to the $\Delta_s + i\Delta_t$ pairing that spontaneously breaks the TRS, and the γ_2 term determines the superconductivity-induced internal magnetic field $\vec{M}_s$ (spin magnetization) by the symmetry constraint $A_2 = A_1 \otimes A_2$, which can be readily detected by the ZF-µSR technique. The minimization of free energy F leads to:

$$M_z = -\frac{\gamma_2}{\alpha_M}\mathrm{Im}[\Delta_s^*\Delta_t].$$

Thus, the superconducting pairings can be expressed as:

$$\Delta(\boldsymbol{k}) = \left[\Delta_s + i\Delta_t\left(d_1 k_x, d_2 k_y, d_3 k_z\right)\cdot\vec{\sigma}\right]\left(i\sigma_y\right).$$

By definition of unitary pairing, Δ_s, Δ_t, and the spin-triplet $\vec{d}(\boldsymbol{k})$ vector are all real. The relative phase between Δ_s and Δ_t ($e^{\pm i\pi} = \pm i$) leads to the spontaneous breaking of time-reversal symmetry. Such pairings were used to analyze the superconducting superfluid density after projecting to the Fermi surfaces upon assuming a weak-pairing limit. Precisely, this energy loss is due to the suppression of Δ_t by the spin-orbit coupling α_R, while the energy gain is given by $-\frac{\lambda_2^2}{\alpha_M}|\Delta_s^* \Delta_t|^2$. Both these two effects are closely related to α_R, the fully-gapped $A_1^s + iA_2^p$ pairing becomes possible if the spin magnetism term wins (i.e., the energy gain is larger than the energy loss). In real materials, this depends crucially on the detailed parameters (e.g., α_R, T_c, etc.).

In a TRS-breaking scenario for a clean system with weak SOC, we also discuss how to distinguish the unitary pairing proposed in this work from a nonunitary pairing. In principle, both pairings can give rise to spontaneous magnetic fields that break TRS in the superconducting state. Nevertheless, in the *T*RuSi NCSC family, the presence of Kramers nodal lines along the Γ-Z direction implies that a unitary pairing would admit only a single-energy gap along such high-symmetry lines. On the contrary, a nonunitary pairing would generally lead to multigap features. To verify which scenario applies to the *T*RuSi family, measurements of, e.g., magnetic penetration depth or upper critical field, with the magnetic field applied along different orientations of *T*RuSi single crystals are in high demand. At the same time, we also have to admit that, due to the multiband nature of *T*RuSi materials, it is not easy to distinguish these states. Another feature of the proposed non-symmetric (s+ip) pairing is the spontaneous breaking of glide symmetry which, in turn, can wipe out the hourglass fermions close to the Fermi energy. Such effects can be used to identify the pairing symmetries.

Note S2

According to two previous theoretical works (Refs. [71,72] in the main text), one can determine the most favorable spin-triplet pairing states in the noncentrosymmetric superconductors with a preserved TRS. This is shown by Eq. (9) in Ref. [71]:

$$\ln(T_c/T_{ct}) = 2\left\langle\left\{\left|\vec{d}(\boldsymbol{k})\right|^2 - \left|\vec{g}(\boldsymbol{k})\cdot\vec{d}(\boldsymbol{k})\right|^2\right\} f(\rho_{\boldsymbol{k}})\right\rangle_{FS},$$

where T_{ct} is the T_c represents the critical temperature where SOC vanishes and the depairing function $f(x) = \mathrm{Re}\left[\sum_{i=1}^{\infty}\left(\frac{1}{2n-1+ix} - \frac{1}{2n-1}\right)\right] \leq 0$ with $\rho_{\boldsymbol{k}} = \alpha_R|\vec{g}(\boldsymbol{k})|/\pi k_B T_{ct}$. Here $\langle\cdots\rangle_{FS}$

represents the integration over the Fermi surface. It proves that only for $\vec{g} \parallel \vec{d}$ at any momenta, T_c is unaffected by SOC. Therefore, an A_1 irrep spin-triplet pairing parallel to the SOC-vector is the most favorable one, i.e., it minimizes the free energy in the superconducting state. As for the other spin-triplet pairings, we usually have $\left|\vec{d}(\boldsymbol{k})\right|^2 - \left|\vec{g}(\boldsymbol{k}) \cdot \vec{d}(\boldsymbol{k})\right|^2 \geq 0$, which means that the superconducting transition temperature T_c is reduced by the presence of SOC, and thus, the pairing strength Δ_t is reduced. As a result, the superconducting free energy increases. However, the direct application of these assumptions to superconductors that spontaneously break TRS is questionable, as currently, the theory has not fully answered this issue. In other words, the TRS-breaking pairing symmetry represents one of the new pairing mechanisms that can coexist with orbital- or spin magnetism, and this is beyond the above theoretical works.

We now discuss the TRS-breaking pairing used in the manuscript. The non-symmetric (*s*+*ip*) pairing can describe the superfluid density quite well (see Fig. 3 in the main text). There are two main reasons for the application of this non-symmetric (*s*+*ip*) pairing to the (Nb,Ta)RuSi superconductors:

(1) Anisotropic SOC

The compounds are anisotropic due to their low crystal symmetry (C_{2v}). Both the effective masses as well as the SOC are different along the *x*- and *y*-directions. As discussed in the above Note S1 for the single-band the effective $\boldsymbol{k} \cdot \boldsymbol{p}$ Hamiltonian is $H_0(\boldsymbol{k}) = \epsilon(\boldsymbol{k})\sigma_0 + \alpha_R \vec{g}(\boldsymbol{k}) \cdot \vec{\sigma}$, where $\epsilon(\boldsymbol{k}) = m_x k_x^2 + m_y k_y^2 + m_z\, k_z^2$ and $\vec{g}(\boldsymbol{k}) = \left(v_1 k_y, v_2 k_x, 0\right)$. To show the anisotropic features on Fermi surfaces, we rescale the momentum and the coefficients for SOC,

$$k_x \to \frac{k_x}{\sqrt{m_x}},\ k_y \to \frac{k_y}{\sqrt{m_y}},\ v_1 \to \sqrt{m_y} v_1,\ v_2 \to \sqrt{m_x} v_2.$$

Thus, the Hamiltonian becomes

$$H_0(\boldsymbol{k}) = \left(k_x^2 + k_y^2 + \mu(k_z)\right)\sigma_0 + \alpha_R\left(v_1 k_y \sigma_x + v_2 k_x \sigma_y\right),$$

where $\mu = m_z k_z^2 - E_F$, with E_F the Fermi energy. Then, the anisotropic SOC is reflected by the difference between v_1 and v_2. Below, we discuss the effect of anisotropic SOC on the spin-triplet $\boldsymbol{d}$-vector.

The A_2 irrep spin-triplet is represented by the $\boldsymbol{d}$-vector $\boldsymbol{d}(k) = (d_1 k_x, d_2 k_y, d_3 k_z)$. According to previous theoretical work (see Ref. [71] in the main text), the d_3 term should vanish. We calculated the superconducting transition temperature T_c/T_{ct} in the spin-triplet channel. Without loss of generality, we set $v_1 > v_2$. And the numerical results are shown in Fig. S14

above. In Fig. S14 (a), the inset shows the two Fermi surfaces in the k_x-k_y space with $k_z = 0$ for $v_1 = 1$ and $v_2 = 0.2$. The calculated T_c is plotted as a function of d_2/d_1. It shows that the $d_2 = 0$ gives a maximum T_c value due to $v_1 \gg v_2$ (i.e., the highly anisotropic SOC). To show the suppression of T_c by SOC, we calculated T_c as a function of v_2/v_1 for $d_2 = 0$. As shown in Fig. S14(b), we find that the increase of v_2/v_1 suppresses the T_c. Therefore, the effect of anisotropic SOC on the A_2 irrep spin-triplet pairing is weak. This conclusion is general and can be directly applied to the (Nb,Ta)RuSi superconductor, where the DFT calculation could confirm $\frac{v_2}{v_1} = 0.1$.

(2) Energy compensation from spin magnetism

According to our previous work (see Ref. [70] in the main text), the $A_1^s + iA_2^p$ can generate an out-of-plane spin magnetism via a third-order coupling term $M_z \mathrm{Im}[\Delta_s \Delta_t^*]$. As also discussed in the Note S1, the free energy of a homogeneous superconductor is given by $F = \alpha_s(T)|\Delta_s|^2 + \alpha_t(T)|\Delta_t|^2 + \alpha_M\left|\vec{M}_s\right|^2 + \gamma_1(\Delta_s\Delta_t)^2 + \gamma_2 M_z \Delta_s \Delta_t + c.c.$. The minimization of free energy leads to $M_z = -\frac{\gamma_2}{\alpha_M}\mathrm{Im}[\Delta_s^*\Delta_t]$. More importantly, the coefficient $\lambda_2 = \frac{7\zeta(3)}{8\pi^3}\frac{\alpha_R k_F}{k_B T_c}$ is proportional to the strength of spin-orbit coupling α_R. Thus, this energy loss is due to the suppression of Δ_t by the spin-orbit coupling α_R, while the energy gain is given by $-\frac{\lambda_2^2}{\alpha_M}|\Delta_s^*\Delta_t|^2$. Both these two effects are closely related to α_R, the fully-gapped $A_{1g}^s + iA_{2g}^p$ pairing becomes possible if the spin magnetism term wins (i.e., the energy gain is larger than the energy loss). Moreover, this (*s*+*ip*) model shows a very good agreement with the superfluid density (see Fig. 3 in the main text).

Considering the above two reasons, we conclude that the non-symmetric (*s*+*ip*) pairing is the most consistent one with the (Nb,Ta)RuSi superconductors in the absence of disorder.
